# *In Silico* Antitubercular Activity Analysis of Benzofuran and Naphthofuran Derivatives

**DOI:** 10.1155/2014/697532

**Published:** 2014-09-11

**Authors:** Prashantha Karunakar, Chamarahalli Ramakrishnaiyer Girija, Venkatappa Krishnamurthy, Venkatarangaiah Krishna, Kunigal Venugopal Shivakumar

**Affiliations:** ^1^Department of Biotechnology, PES Institute of Technology, BSK III Stage, Bangalore 560085, India; ^2^Department of Chemistry, SSMRV College, Jayanagar 4th T Block, Bangalore 560 041, India; ^3^Department of Biotechnology and Bioinformatics, Kuvempu University, Shankaraghatta 577451, India

## Abstract

For the human health, *Mycobacterium tuberculosis* (MTB) is the deadliest enemy since decades due to its multidrug resistant strains. During latent stage of tuberculosis infection, MTB consumes nitrate as the alternate mechanism of respiration in the absence of oxygen, thus increasing its survival and virulence. NarL is a nitrate/nitrite response transcriptional regulatory protein of two-component signal transduction system which regulates nitrate reductase and formate dehydrogenase for MTB adaptation to anaerobic condition. Phosphorylation by sensor kinase (NarX) is the primary mechanism behind the activation of NarL although many response regulators get activated by small molecule phospho-donors in the absence of sensor kinase. Using *in silico* approach, the molecular docking of benzofuran and naphthofuran derivatives and dynamic study of benzofuran derivative were performed. It was observed that compound Ethyl 5-bromo-3-ethoxycarbonylamino-1-benzofuran-2-carboxylate could be stabilized at the active site for over 10 ns of simulation. Here we suggest that derivatives of benzofuran moiety can lead to developing novel antituberculosis drugs.

## 1. Introduction

Furan having various pharmacological and biological activities such as antituberculosis [1], anti-inflammatory [2], and antibacterial [3] activities attracted the attention of synthetic chemists. Antitubercular activity has been observed [4] in* in vitro* analysis using resazurin assay by microtiter-plate method (REMA) of benzofuran derivatives. Substituted benzofurans find their applications in different fields such as fluorescent sensors [5], antioxidants, brightening agents, and a variety of drugs and agriculture.

In the major global infectious diseases,* Mycobacterium tuberculosis *(MTB) is second after HIV/AIDS. 1.4 million people died and 8.7 million people fell ill from TB during 2011. In low-income and middle-income countries over 95% of TB deaths occur and it is among top three causes of death for women aged from 15 to 44 [6]. The availability of nutrients in the host and their metabolism by MTB plays a major role in mycobacterial pathogenesis and persistence during anaerobic condition. Due to multidrug-resistant tuberculosis (MDR-TB), bacteria could not respond to either isoniazid or rifampicin, the two most powerful, first-line or standard anti-TB drugs. Mycobacteria can adapt to the host environment by two-component signal transduction system (TCS), where they sense, respond, and adapt to changes in the environment by modulating the expression of subsets of genes in response to specific extracellular signals.

There are 11 complete TCSs in MTB [7]. A prototypical two-component system consists of a histidine protein kinase (HK) and a response regulator (RR). RRs have a modular architecture consisting of a conserved receiver domain and a variable effector domain consisting of DNA binding elements [8]. NarX-NarL and NarQ-NarP are two-component systems that are involved in the regulation of anaerobic respiratory gene expression in response to nitrate and nitrite [9]. NarX is a membrane-bound nitrate sensor [10] and NarL is a cytoplasmic response regulator consisting of N-terminal receiver domain and C-Terminal effector domain. NarL is involved in the synthesis of nitrate reductase (encoded by narGHJI) and formate dehydrogenase-N (encoded by fdnGHI operon) in the presence of nitrate and these enzymes are involved in nitrate respiration [11]. NarQ is a sensor kinase which can independently sense the presence of nitrate and transfer the signal to NarL. Furthermore, small molecule phosphor-donors such as acetyl phosphate can also phosphorylate NarL* in vitro* [12]. The phosphorylation-dependent activation of NarL exposes the C-Terminal domain for binding to the DNA to activate or repress the transcription [13]. During anaerobic growth, nitrate acts through NarL to activate and repress the anaerobic respiratory gene expression [9]. The gene narL coding for NarL protein is upregulated four-fold in the late stationary phase cultures of MTB [14]. There is 35% overall amino-acid identity between* E. coli *and MTB NarL and their active sites superimpose exactly and the positions of the active site residues are nearly identical [30]. Based on this sequence homology, NarL has been predicted to be involved in the regulation of anaerobic nitrogen metabolism in MTB (Tuberculist website: http://genolist.pasteur.fr/TubercuList/) and is identified as potential drug target (http://www.uniprot.org/uniprot/O53856). Blocking the active site will inhibit the phosphorylation activity of NarL and negatively affects the MTB adaptation.

## 2. Materials and Methods

Protein-ligand interaction of ethyl naphtho[2,1-b]furan-2-carbothioate (C1), 2-naphtho[2,1-b]furan-2-yl-5-phenyl-1,3,4-oxadiazole (C2), ethyl 8-nitronaphtho[2,1-b]furan-2-carboxylate (C3), 1-(2-chlorophenyl)-1-(8-nitronaphtho[2,1-b]furan-2-yl)urea (C4), (4-nitrophenyl)-1-(8-nitronaphtho[2,1-b]furan-2-yl)urea (C5), ethyl 3-amino-5-bromo-1-benzofuran-2-carboxylate (C6), ethyl 5-bromo-3-ethoxycarbonyl amino-1-benzofuran-2-carboxylate (C7), and ethyl naphtho[2,1-b]furan-2-carboxylate (C8) with protein target NarL of* Mycobacterium tuberculosis* was carried out to understand its antitubercular activity.

### 2.1. Antitubercular Activity of Benzofuran and Naphthofuran Derivatives

For the molecular docking analysis, newly synthesized furan derivatives were considered to analyze the antitubercular activity. In these synthesized furan derivatives, two compound structures were solved crystallographically, mainly C6 [15] and C7 [16]. The three-dimensional atomic coordinates of C6 and C7 were prepared using WinGX [17]. Since the conformation of the benzofuran and naphthofuran derivatives have been determined from single crystal X-ray diffraction, it is worthy to look at their binding pattern to obtain meaningful insights for these experimentally determined conformations. These studies also provide inputs for drug design which reveal conformational variant at the active site of protein [18]. The coordinates of these compounds along with other derivatives were further extrapolated for docking studies.

### 2.2. NarL Structure

**Figure 1:**
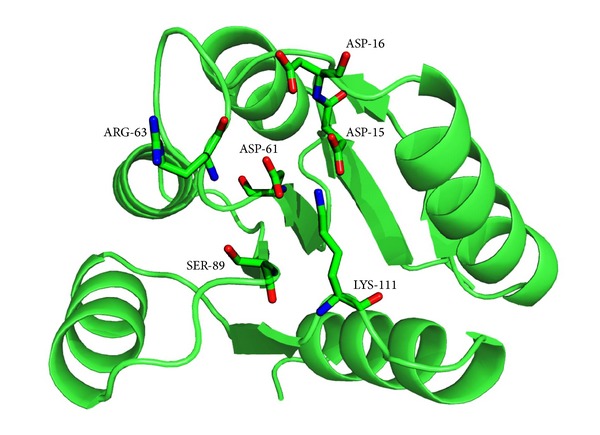
Active site residues of NarL are represented in ball and stick.

The NarL protein (in the asymmetric unit) is a homotetramer containing 4 identical subunits having their independent active sites each for subunits A, B, C, and D, respectively. This structure represents the N-terminal signal receiver domain containing 4 identical chains. The coordinates of crystal structure of 1.9 Å of the signal receiver domain of the putative response regulator NarL from* Mycobacterium tuberculosis* (PDB ID: 3EUL) was obtained from the Protein Data Bank. Though protein is homotetramer, the crystallographer determined biologically active monomeric form was considered for the docking and dynamics simulation study.  Figure 1 shows the active site residues Asp15, Asp16, Arg63, Ser89, and Lys111 and site of phosphorylation Asp-61 [30]. Schnell et al. have indicated that from native PAGE analysis and the elution profile in size exclusion chromatography and gel-filtration chromatography, NarL-N is a monomer in solution and it migrated in the native gel as a single well-defined band. Hence, biologically active unit has been considered for docking and molecular dynamics.

### 2.3. Molecular Docking Processing

Topology file and other force field parameters were generated for ligand using the PRODRG program [19]. The Kollman charges were added to the protein atoms and the Gasteiger partial charges were assigned to the ligand atoms using the program AutoDock. All the ligands were converted to  .pdbqt format using OpenBabel tool built inside PyRx 0.8 docking tool. Flexible torsions for all ligands were defined using AUTOTORS. The docking site for the ligands on 3EUL was defined at the active site with grid box size 48 × 56 × 48, spacing 0.375, and grid centre −9.584, 3.945, and 15.45. The autogrid module calculated the electrostatic map and atomic interaction maps for all atom types of the ligand molecules. The Lamarckian Genetic Algorithm (LGA) was selected with the population size of 150 individuals and with a maximum number of generations and energy evaluations of 27,000 and 2.5 million, respectively. Default parameters like elitism (1), mutation (0.02), and crossover rate (0.8) were considered. From the estimated free energy of ligand binding (Δ*G*), the inhibition constant (*K*_*i*_) for the ligands was calculated. Results from the AutoDock run were analyzed using molecular graphics laboratory (MGL) tools [20] and the best conformation with least binding energy interaction was analyzed using Ligplot^+^ [21]. PyMOL was used for docking conformation representation [22].

To overcome any serendipity, positive controls such as acetyl phosphate, carbamoyl phosphate, dihydroxyacetone-P, phosphoramidate, and monophosphoimidazole were used throughout the docking procedure. Interaction profile for natural phospho-donors is included in Table 1.

### 2.4. Molecular Dynamics

Molecular dynamics simulation was carried out to study the real time interaction and stability of the top scoring compounds in the biological environment. The study also gives the detailed description of the molecular interactions in the solvent environment, which is very close to reality. Molecular dynamics simulation was carried out for 10 ns using Gromacs version 4.5.5.

### 2.5. Premolecular Dynamics Processing

The system contained protein, water molecules, and appropriate number of sodium ions all enclosed in a periodic box. Hydrogens and charges were added and protein was defined using Amber-99SB force field parameters.

Using UCSF chimera visualization tool [23], protein was prepared for dry run by explicitly adding hydrogens and AM1-BCC partial charges. Protein was defined using Amber-99SB force field parameters [24]. Ligand was defined using generalized amber force field (GAFF) parameters [25] and AM1-BCC partial charges were added using ANTECHAMBER [26] followed by conversion to GROMACS compatible topology using ACPYPE [27]. Complex was prepared for production run using chimera.

### 2.6. Molecular Dynamics Simulation

**Figure 2:**
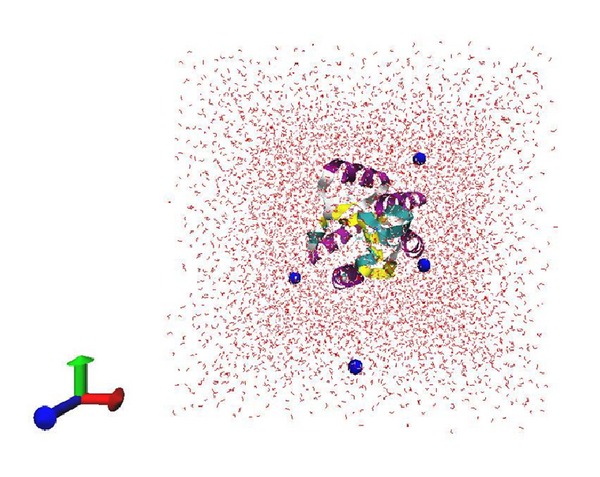
Neutralization of system by addition of four sodium ions. Stick model represents the water molecules and ball model represents NA ions (Blue) and protein is shown as ribbon enclosed inside the box with secondary structure coloring.

All MD simulations were performed using GROMACS version 4.5.5 compiled in single-precision mode [28, 29]. A simulation cell was created in a cubic periodic box with a minimum distance of 10 Å between the protein and the box walls. The protein was bathed with TIP3P water molecules along with four sodium ions to neutralize the system (Figure 2).

Energy minimization was performed by using 5000 steps of steepest descent coupled with conjugate gradient method at every 100 steps or until the maximum force was smaller than 100 kJ mol^−1^ nm^−1^. The electrostatic interactions, particularly of long range were calculated using Particle-Mesh Ewald method (PME). The cutoff for distance was 1 nm. The dispersion interactions, both short-range repulsive and attractive, as described by Lennard-Jones, had a cutoff of 1 nm. The LINCS algorithm was used to constrain bonds allowing a time step of 1 fs. At every 10 steps, neighbor searching was carried out. A Parrinello-Rahman barostat pressure of 1 bar was used with a coupling constant of Tau_P = 0.5 ps and compressibility of 4.5e-5 (bar^−1^). Water, protein, ions, and ligand molecules were coupled separately to the thermal bath at 300 K, using a v-rescale coupling constant Tau_T = 0.1 ps. An initial preparatory run for 500 ps was carried out to allow the randomization of water molecules around the protein. The position restrained molecular dynamics was carried out using mdrun program. The isobaric-isothermal ensemble simulation was performed for 10 ns. The results were analyzed in VMD tool and Gromacs.

## 3. Results and Discussions

### 3.1. Molecular Docking Studies of Benzofuran and Naphthofuran Derivatives

**Figure 3:**
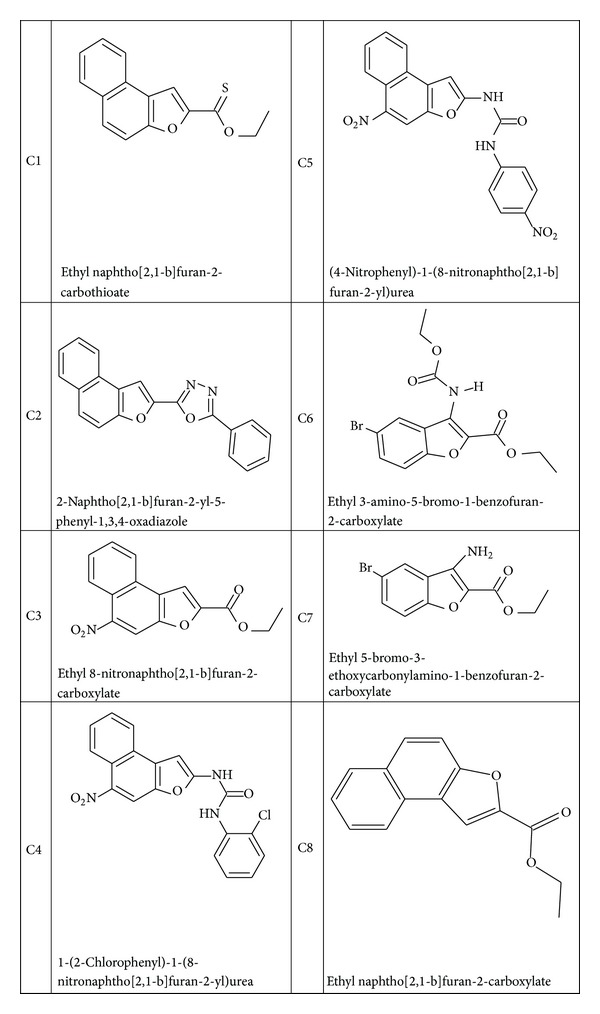
Benzofuran and naphthofuran derivatives structures used in docking studies.

Chemical structures of the benzofuran and naphthofuran derivatives considered for docking are listed in Figure 3.

### 3.2. Antitubercular Activity Study of Benzofuran and Naphthofuran Derivatives

The* in silico *interaction of phospho-donors to NarL was carried out and it was observed that all compounds were binding to active site, though blind docking was performed. All the natural phospho-donors (acetyl phosphate, carbamoyl phosphate, phosphoramidate, and monophosphoimidazole) bind to active site with binding energy ranging from −3.32 to −3.93 kcal/mol and inhibition constant from 1.31 mM to 3.67 mM (Table 1).

**Figure 4:**
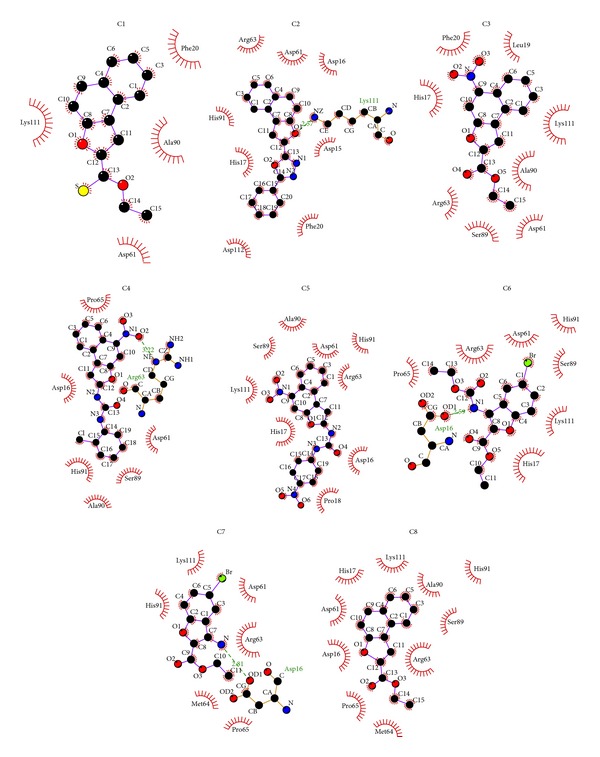
2D representation using Ligplot^+^ of all compounds interacting at the active site of NarL. Hydrogen bonds are represented by dashed lines.

The conformation with least binding energy and most stability based on cluster analysis was taken for docking analysis. The docking interaction details are represented in Table 2. Out of 8 compounds docked, C1 shows least binding energy of −6.99 kcal/mol and C3 with highest binding energy of −3.76 kcal/mol. From the estimated inhibition constant it is observed that micromolar concentration of all compounds is sufficient to inhibit the protein activity by 50% (IC_50_). The less quantity of 7.54 mM is required to have desired IC_50_ effect when compared to C7 which requires 940 mM. From the Ligplot^+^ diagrams (Figure 4), it is observed that compounds C1, C3, C5, and C8 show a hydrophobic interaction. One N-H*⋯*O hydrogen bond was observed in C2, C4, C6, and C7 at the active site of NarL. Either the nitrogen of ligand or nitrogen of active site residue is involved in bond formation. Compounds C6 and C7 formed hydrogen bond with site of phosphorylation residue Asp16, bromine atom, and carboxylate group showing hydrophobic interaction at the active site. C2 and C4 hydrogen bonded with Lys111 and Arg63, respectively. Similar N-H*⋯*O intermolecular hydrogen bonding was observed in crystal packing of C6 and C7 [15, 16].

**Figure 5:**
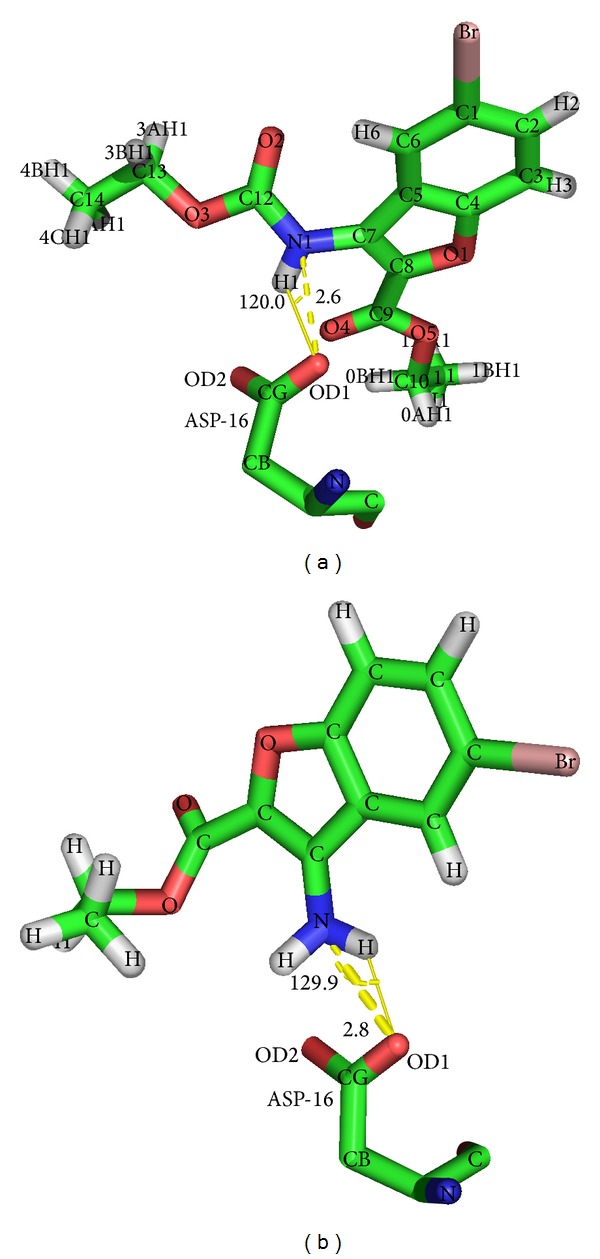
N-H*⋯*O intermolecular hydrogen bonding of C6 (a) and C7 (b) compared with crystal packing.

In C7, intermolecular N1-H1A*⋯*O2 (symmetry code: *x* + 1,  −*y* + 1/2,  *z* + 1/2) was observed with bond distance of 2.977 (4) Å and angle of 171 (3)°. However, the result of docking correlates more with intramolecular hydrogen bonding N1-H1B*⋯*O3 with bond distance of 2.835 (4) Å and angle of 123 (3)°. The hydrogen bonding length and angles of C6 and C7 are represented in Figure 5. The hydroxyl group appears to hold the strong intramolecular hydrogen bonded feature as observed in the crystal structure of ligand. Though binding energy, inhibition constant are used as important indicators in molecular docking studies, they cannot offer complete characteristics of the interaction in all the cases, thus the use of dynamic simulation becomes inevitable.

**Figure 6:**
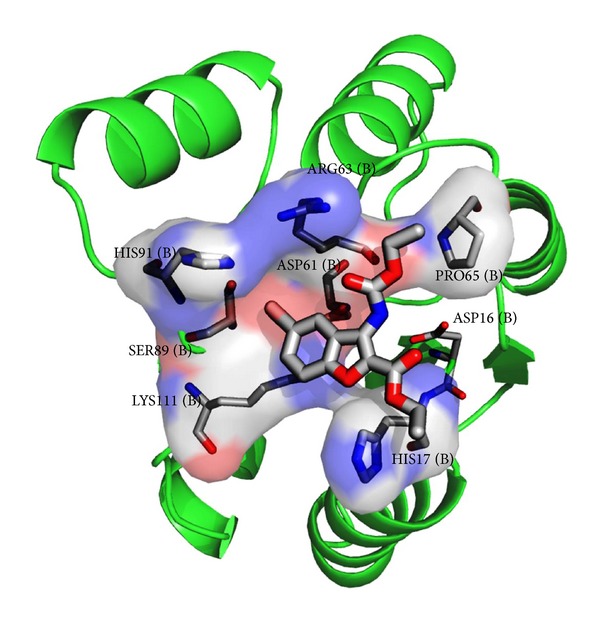
Interaction of C6 at the active site of NarL.

**Figure 7:**
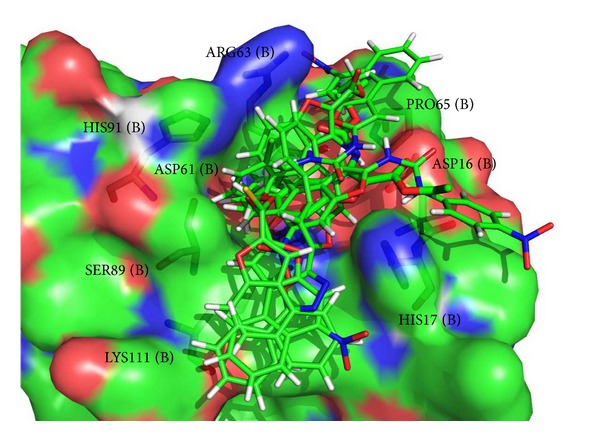
Interaction of all ligands at the active site of NarL.

Figure 6 shows the molecular docking interaction of compound 6 alone as it was further extrapolated to dynamics simulation.  Figure 7 depicts the interaction of all ligands at the active site of NarL. This suggests that these groups are responsible for stabilizing the interactions in the active site and blocking the site of phosphorylation of NarL.

### 3.3. Molecular Dynamics Simulation Analysis

**Figure 8:**
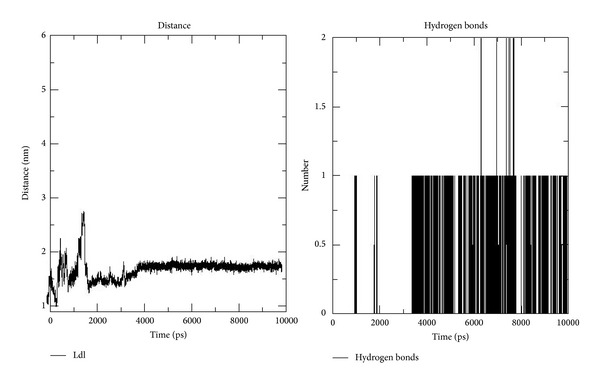
Distance plot of C7 from the active site residue Asp-61 through the 10 ns.

**Figure 9:**
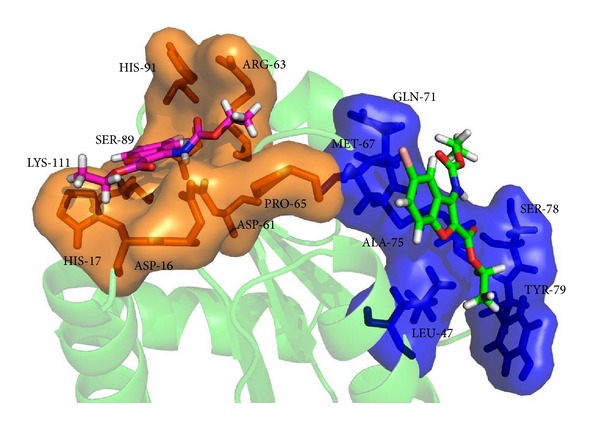
Movement of ligand C7 interaction from pocket-1 (orange) to pocket-2 (blue).

**Figure 10:**
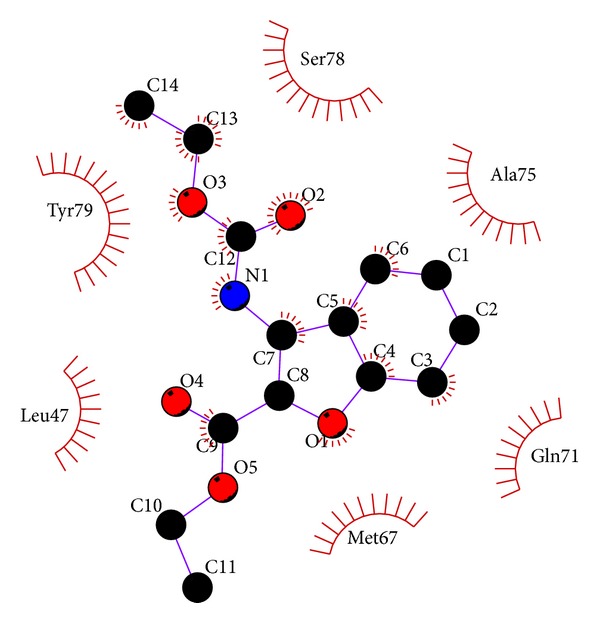
Interaction of C7 in the active site of NarL after molecular dynamic simulation.

The crystallographically solved (C7) structure was further extrapolated to dynamics studies. The molecular dynamics simulation showed hydrogen bonding for most parts of 10 ns run (Figure 8). Though the compound was fixed at the active site (Pocket-1), it moved to another (pocket-2) and interacted with Leu47, Met67 and Gln71, Ala75, and Ser78 and Tyr79 (Figure 9). There is a local structural difference at the loop region and *α*-helix region from 64 to 77 in both NarL of* M. tuberculosis *and* E. coli*. The residue Met67 has the maximal displacement of 7.7 Å in Cα position as compared to NarL of* M. tuberculosis* and* E. coli. *Even from the crystallography it is unclear whether this loop region difference is due to crystal lattice or it reflects any biological relevant loop conformation [30]. This movement could be due to less steric hindrance with Ala 44, Met 67, and Ala 74-75 and aiding the ligand to be in the hydrophobic pocket and the remaining part of the ligand to be in the polar region of the protein.  Figure 10 represents the interaction of C7 in the active site of NarL after molecular dynamic simulation.

## 4. Conclusion

From the docking study, it can be noticed that the crystallographically determined structures C6 and C7 possess a bromine atom along with the benzofuran ring. It showed that the hydrophobic interaction at the active site and the nitro group forms hydrogen bonding with aspartic acid 16, thereby stabilizing the complex. From the molecular dynamics simulation study on the compounds, it can be suggested that all benzofuran and naphthofuran derivatives have affinity to interact at the active site of NarL and have the potential to act as lead molecules. From the molecular dynamics study, it was observed that local structural difference at the loop and α-helix regions from 64 to 77 residues is flexible for the movement of ligand towards Met67. This movement could be due to less steric hindrance with Ala 44, Met 67, and Ala 74-75 and aiding the ligand to be in the hydrophobic pocket and the remaining part of the ligand to be in the polar region of the protein. Also, there is no hydrogen bonding between the ligand and the NarL protein. Another crystallographically determined structure C8 shows only hydrophobic interaction in the docking studies.

From the docking analysis of naphthofuran and benzofuran derivatives, it can be concluded that in most of the interactions nitro group formed hydrogen bond with Asp16, bromine atom, and carboxylate group showing hydrophobic interaction at the active site. Binding energy and inhibition constant are also minimal showing that these compounds have high affinity towards NarL protein. This suggests that these groups are responsible for stabilizing the interactions in the active site, thereby blocking the site of phosphorylation of NarL. Hence, these compounds may act as a potential antitubercular lead molecule.

## Figures and Tables

**Table 1:** Energy profile of small molecule phospho-donors.

SI number	Compound	AutoDock 4.2 binding energy (kcal/mol)	Inhibition constant
1	Acetyl phosphate	−3.93	1.31 mM
2	Carbamoyl phosphate	−3.82	1.58 mM
3	Dihydroxyacetone-P	−3.32	3.67 mM
4	Phosphoramidate	−3.84	1.54 mM
5	Monophosphoimidazole	−3.82	1.58 mM

**Table 2:** Molecular docking interaction details of benzofuran and naphthofuran derivatives against NarL protein.

Compounds	BE∗	LE∗	IC^+^	IE∗	VE∗	EE∗	TI∗	TE∗
C1	−6.99	−0.39	7.54	−7.88	−7.82	−0.06	0.2	0.89
C2	−5.26	−0.22	140.42	−5.85	−5.63	−0.22	−0.39	0.6
C3	−3.76	−0.18	1750	−5.25	−5.37	0.12	−0.32	1.49
C4	−5.14	−0.19	171.81	−6.03	−5.93	−0.1	−0.8	0.89
C5	−4.31	−0.15	696.53	−5.5	−5.55	0.05	−0.43	1.19
C6	−5.21	−0.25	152.05	−7.6	−7.6	0	0	2.39
C7	−4.13	−0.26	940.59	−5.62	−5.35	−0.27	−0.18	1.49
C8	−4.41	−0.25	582.62	−5.31	−5.5	0.2	−0.11	0.89

BE = binding energy, LE = ligand efficiency, IC = inhibition constant, IE = intermolecular energy, VE = Van der Waals + hydrogen bonding + desolvation energy, EE = electrostatic energy, TI = total internal energy, TE = torsional energy, ∗ = kcal/mol, and + = micro molar.
